# Blunted startle reactivity in everyday sadism and psychopathy

**DOI:** 10.1038/s41598-023-41043-2

**Published:** 2023-08-30

**Authors:** Erin E. Buckels, Douglas A. Williams, Paul D. Trapnell, Siavash Kermani Koosheh, Owen M. Javra, Sasha C. Svenne

**Affiliations:** Department of Psychology, University of Winnipeg, 515 Portage Avenue, Winnipeg, MB R3B 2E9 Canada

**Keywords:** Human behaviour, Behavioural methods

## Abstract

Two studies examined the amplitude of the startle response as a function of the Dark Tetrad of personality (narcissism, Machiavellianism, psychopathy, and sadism). We measured electromyographic activity of the *orbicularis oculi* muscle evoked by a startle stimulus while participants viewed images on a computer screen. Both studies revealed a negative correlation between general startle reactivity (averaged across positive, negative, and neutral images) and sadistic tendencies. In Study 2, all four dark traits were negative correlates of general startle reactivity. Study 2 also examined the personality correlates of aversive startle potentiation (ASP; indexed by greater reactivity while viewing negatively-valenced images than positive or neutral images). ASP correlated negatively with a variety of personality measures of psychopathy and sadism, their facets, and related personality tendencies (callousness, risk-taking, and restricted affect). These findings suggest that ordinary people with high levels of callousness and antagonism display physiological evidence of non-reactivity (i.e., blunted acoustic startle in general), whereas psychopathy and sadism are preferentially associated with reduced ASP.

## Introduction

Most people avoid hurting innocent others. For some, however, inflicting suffering may generate a sense of pleasure or a feeling of excitement. This appetite for cruelty, *sadism*, is part of a constellation of personality dispositions known as the Dark Tetrad^1–6^, which includes three other notorious and socially-aversive traits: Narcissism, characterized by grandiose self-promotion; Machiavellianism, characterized by strategic duplicity and manipulation; and psychopathy, characterized by reckless misconduct without remorse. The Dark Tetrad share a propensity for callous exploitation and antagonism^7, 8^, and are moderately to strongly intercorrelated^5^, a psychometric overlap sometimes referred to as “D”, the dark core^9, 10^. Despite their overlap, these four traits are empirically and conceptually distinguishable from one another^5, 8, 11, 12^. For example, while unprovoked aggression is associated with all members of the Dark Tetrad^13, 14^, sadism is the only unique predictor of willingness to work to hurt innocent people^1^. Narcissism, Machiavellianism, and psychopathy also show unique associations with construct-relevant antisocial behaviors: for example, narcissism is the strongest predictor of aggression following an insult^15^; Machiavellianism is the best predictor of effort in designing online scams^16^; and psychopathy is the sole predictor of cheating in a high-risk scenario^17^. These association patterns highlight the distinct nature of the Dark Tetrad traits and the importance of assessing them concurrently in psychological research.

Theorizing on the biological basis of antisocial traits from an evolutionary perspective may explain some of the similarities and differences among the Dark Tetrad^18^. Twin studies reveal high heritabilities for narcissism and psychopathy, and a moderate heritability for Machiavellianism, which also has a shared environmental component unlike the others^19^. No data are currently available for sadism. One explanation for these twin study findings is dark traits offer alternative survival and mating strategies for small subgroups within a highly social human species. Evolutionary psychologists have speculated on how narcissism^20^, Machiavellianism^21, 22^, and psychopathy^23^ may enable some to enhance their own individual fitness at the expense of others. As for sadism, being cruel and aggressive against outgroups may benefit sadistic men with opportunities for reproduction and social status within their group^24^. Predatory aggression in war-experienced soldiers who have overcome fear seems to have appetitive hedonic elements^25^, which may explain the vindictive demeanor of those high in sadistic traits^26, 27^. Sadism also requires attenuated negative emotional functioning to facilitate violent behavior and enjoyment^28^. Like a cat stalking a bird, violence finds expression in the absence of empathy.

One psychophysiological marker that might distinguish the four components of the Dark Tetrad is the startle response^29–31^. Startle is readily measured in the laboratory by monitoring the movement of the *orbicularis oculi* muscle surrounding the orbit of the eye^32^. Sudden threatening stimuli evoke a protective eye wince and gross body movements as defensive reactions. Studies examining startle reactivity often include manipulations to alter the participant’s emotional state^33^. *Aversive startle potentiation* (ASP) refers to the enhancement of startle while participants are experiencing negative emotions (e.g., disgust, fear, sadness), which is most often accomplished in the laboratory by having people view negative images or movie clips. *General startle* (GS) is a baseline measure independent of experimental manipulations. Both indexes provide meaningful information about chronic startle tendencies, which may be elevated in some clinical populations and reduced in others. For example, individuals with post-traumatic stress disorder show strong startle reactivity in both dangerous and safe contexts^34^.

Two overlapping populations reliably linked to blunted startle are repeat offenders and persons with elevated psychopathic traits^35^. In contrast to those suffering from trauma, the startle response of criminal psychopaths is minimal and unrelated to the emotional valence of images shown during startle testing^36–38^. Considerable evidence exists that reduced ASP is uniquely associated with Factor 1 scores on the Psychopathy Checklist-Revised^39^ in incarcerated men^31, 38, 40^, incarcerated women^41^, and non-forensic samples^42^. This interpersonal factor includes tendencies of shallow affect, callousness, and manipulation/exploitation. Factor 2 traits such as impulsiveness and irresponsibility are often not predictive of diminished startle.

Sadism is closely related to psychopathy in forensic populations^43, 44^. These traits have common antisocial behavioral elements^3^ and are characterized by a lack of empathy^45^. Research on sadism in clinical-forensic populations has focused on the enjoyment of illegal activities causing serious bodily harm, such as physical violence and sexual predation^46–51^. Sadism, like the other traits comprising the Dark Tetrad, can also be found in non-forensic samples. Subclinical or everyday sadism is characterized by the enjoyment of watching others in pain and engaging in aggressive acts, such as internet trolling^52^ or playing violent video games^53–55^. Everyday sadists will pay a price in the laboratory to engage in disturbing activities, such as placing a live bug in a grinder^1^, which suggests these behaviors are intrinsically reinforcing. Both psychopathy and sadism correlate with schadenfreude^56, 57^, which is pleasure from another’s misfortune without directly causing it^58^. Sadism also predicts enjoyment of violent videos independently of overlap with the other Dark Tetrad traits^59^.

Given its close relationship with psychopathy^60, 61^, sadism may also predict reduced startle reactivity. Sadism requires callousness^8^, a significant factor in diminished startle potentiation by violent imagery^62^. Sadistic pleasure, a socially-deviant positive emotion, may decrease startle reactivity when viewing negative images that are upsetting or disturbing to others. This prediction is based on the well-established effects of positive affect in buffering against stress^63^ and reducing startle reactivity^33^. Therefore, due to the unique combination of callousness and pleasure from suffering, sadism may predict both blunted ASP and blunted startle reactivity in general. To our knowledge, no previous research has investigated this relationship while controlling for related traits that are reliably linked to startle reactivity (e.g., psychopathy). Additionally, no research has examined blunted startle associations with the Dark Tetrad of personality when using a measurement approach that minimizes the psychometric overlap between these traits, a longstanding issue recently addressed with the development of the Short Dark Tetrad (SD4) measure^5, 12^. We aimed to address these gaps in the literature.

## Overview of the studies

The purpose of the two studies described here was to examine the relationship between the Dark Tetrad and blunted startle reactivity (both affectively modulated and general startle). Participants underwent startle testing after completing a battery of questionnaires to assess their personalities. In both studies, startle probes were delivered while participants viewed emotional images depicting pleasant/interesting, neutral/dull, or unpleasant/disturbing scenes. Study 1 examined personality associations with general startle reactivity averaged across all image valences. In Study 2, we arranged the images into movie-like sequences, permitting us to examine personality associations with ASP.

Across these studies, we expected a negative relationship between startle reactivity and the Dark Tetrad traits, especially psychopathy and sadism. Given the shared callousness between psychopathy and sadism, it was expected that both traits would exhibit negative associations with startle reactivity. However, considering their distinct characteristics, such as psychopathic boldness^64^ and sadistic pleasure from cruelty^1, 8^, we also anticipated unique associations when accounting for the overlap with the other trait and mean gender differences. To test these predictions, we administered multiple measures of sadism and psychopathy, emphasizing the SD4 questionnaire, which offers superior discrimination between these traits^5, 12^. The SD4 scales served as the primary focus of our analyses, complemented by standalone measures of sadism^65, 66^ and psychopathy^64, 67^, including multidimensional instruments in Study 2. By incorporating multiple measures of the same constructs, our goal was to triangulate the true associations with startle reactivity.

Recognizing that anxiety levels influence startle reactivity^68, 69^, we also included several scales to measure anxiety and related tendencies^70–72^. In Study 2, we selected facets from the Personality Inventory for DSM-5^73, 74^ to index specific maladaptive traits involved in sadism and psychopathy (i.e., callousness, hostility, restricted affect, and risk-taking) and negative affect (i.e., anxiousness and emotional lability) that might influence startle responses. The inclusion of measures related to anxiety and maladaptive tendencies offered valuable context for understanding the associations between Dark Tetrad traits and startle reactivity, while potentially providing insights into the underlying mechanisms of these effects.

This research was approved by the Human Research Ethics Board at the University of Winnipeg, and all experiments were performed in accordance with Canada’s Tri-Council Policy for Ethical Conduct for Research Involving Humans.

## Study 1

### Method

#### Participants

Two groups of undergraduate students (Total *N* = 160) were recruited to participate in the study in exchange for partial course credit. An a-priori power analysis^75, 76^ indicated that a minimum sample size of 123 participants was necessary to detect a moderate correlation of ρ = 0.25 with power (1 – β) = 0.80. All participants were required to have normal or corrected vision and normal hearing. Cohort 1 included 94 (71 women, 24 men) participants drawn from the subject pool without preselection. Cohort 2 included 66 participants (47 women, 19 men) preselected from the lowest or highest sadism quintile on the SD4 (included in a separate survey administered in September 2018). Data from Cohort 2 maximized the sadism variance in the overall sample. Startle testing occurred from December 2018 to March 2019. Informed consent was obtained from all participants.

#### Measures

##### Anxiety Sensitivity Index (ASI-3)^70^

The Anxiety Sensitivity Index assesses apprehension about anxiety symptoms. This 18-item scale has three subscales covering physical, cognitive, and social anxiety symptoms, with items rated on a 5-point Likert scale from 0 (*very little*) to 4 (*very much*). Cronbach’s alphas ranged from 0.75 to 0.90.

##### Behavioral Inhibition System/Behavioral Activation System (BIS/BAS) scale^71^

The BIS/BAS measures aversive and appetitive motivation. The scale has 24 items with a single BIS subscale (motivation to avoid negative/painful outcomes) and three BAS subscales (motivations to approach). Statements were rated on a 4-point Likert scale from 1 (*very true for me*) to 4 (*very false for me*). Cronbach’s alphas ranged from 0.67 to 0.80.

##### Intolerance of Uncertainty Scale-Short Form (IUS-12)^72^

The IUS-12 is a short 12-item version of the Intolerance of Uncertainty Scale^77^ that measures responses to uncertainty, ambiguous situations, and the future. The items were rated on a 5-point Likert scale ranging from 1 (*not at all characteristic of me*) to 5 (*entirely characteristic of me*). Cronbach’s alpha was 0.89.

##### Self-Reported Startle (SRS)

During the subject pool registration process, participants estimated their subjective startle response with a single item, “*Are you easily startled?,*” on a 5-point Likert scale with response options, 1 (*Not at all*), 2 (*A little bit*), 3 (*Somewhat*), 4 (*Very easily*), and 5 (*Extremely easily*). The item also appeared in the questionnaire battery administered before startle testing. The two ratings were positively correlated (*r* = 0.45) and were averaged to compute an overall measure of Self-Reported Startle (SRS).

##### Short Dark Tetrad (SD4)^5^

The SD4 is a brief screening measure for Machiavellianism, narcissism, psychopathy, and sadism. It is the only instrument that permits concurrent assessment of the Dark Tetrad traits with maximum discrimination, making it ideal for the current investigation. We administered this measure twice: the first administration was done online and used only to identify the top and bottom quintiles; the second was conducted in person, alongside the other questionnaire measures, and was used to predict behavioral startle. The 28 items were rated on a 5-point Likert scale from 1 (*strongly disagree*) to 5 (*strongly agree*). Cronbach’s alphas ranged from 0.72 to 0.87.

##### Short Sadistic Impulse Scale (SSIS)^65^

The SSIS, a 10-item measure of subclinical sadism, shares similarities with the SD4 sadism scale as both instruments provide brief assessments of overall sadistic tendencies. Consequently, we chose to include the SSIS as a complement to the SD4 measure in our study. Items were rated on a Likert scale from 1 (*strongly disagree*) to 5 (*strongly agree*) in Study 1. Cronbach’s alpha was 0.88.

#### Procedure

Upon entering the laboratory, the participants completed a series of questionnaires measuring anxiety (ASI-3, BIS/BAS, and IUS-12) and dark personality (SD4 and SSIS). Questionnaires were administered on an iMac computer (Superlab 5.0, Cedrus Inc., San Pedro, CA), which was also used for startle testing. Startle preparation and testing occurred immediately after the completion of the questionnaires.

##### Startle testing

Two disposable electrodes (Vermed A-100040, Buffalo, NY) were placed over the *orbicularis oculi* muscle under the right eye. One electrode was placed in line with the pupil, a second electrode was placed about 1.5 cm to the right, and a ground electrode was placed in the middle of the forehead. Startle stimuli included a 200-ms air puff (17 psi) delivered just above the participant’s eyebrows via a 1 mm hole in an otherwise closed plastic tube that ran across the top of the frame of a pair of glasses. The air compressor was in an adjacent room. A 40-ms burst of white noise with an instantaneous rise time (102 dB: Scale A), presented binaurally through stereo headphones, served as the other startle stimulus. Participants experienced the two startle stimuli once each before the study began. Startle magnitude depends on both fear and general anxiety, the latter being affected by unpredictability^78^. It follows that using both tactile and auditory startle stimuli might result in a heightened startle response because participants would be unable to anticipate the specific type of startle probe they would experience on the trial.

Electromyography data were collected using a commercially available startle response monitoring system (SR-HLAB, San Diego Instruments, San Diego, CA). Startle testing was conducted while participants viewed emotional images on a desktop computer (Superlab 5.0, Cedrus Inc., San Pedro, CA), which triggered a laptop in an adjacent room. The laptop recorded electromyography data and controlled the delivery of the startle stimuli. We informed participants that they would see a series of images with varying content, some of which may be difficult to look at. We asked them to attend to the images for the entire viewing period and ignore any air puffs on their forehead or noises over the headphones. At the end of the study, there would be a recognition test. The purpose of this procedure was to encourage participants to fully engage with the images, even if they found them unpleasant.

The 48 images used in Study 1 (see Table S1 in the supplemental materials) were drawn from the International Affective Picture System (IAPS)^79^, and divided into two sets of 24 images (counterbalanced). One set was shown during both startle and recognition testing, and the other set appeared during the later recognition test as lures. During startle testing, the participants viewed 24 IAPS images for 4 s each, with an intertrial interval varying from 16 to 24 s. The images were grouped into two blocks of 12 images—each block consisting of three negative images (IAPS mean valence = 2.00), three neutral images (IAPS mean valence = 5.00), and three positive images (IAPS mean valence = 7.94). There were also three startle probes presented during the intertrial interval in the absence of a photo. One of the two startle stimuli was presented on each trial, either 1.2 s or 3.2 s after slide onset. Image valence (positive, neutral, negative, and no photo), startle stimulus (tactile or auditory), and onset time (short or long) were varied using a Latin square counterbalancing procedure. Participants received a total of 32 startle probes, consisting of 16 tactile (air puff) and 16 auditory (noise) stimuli. After startle testing, the participants received a memory test with the 24 images seen during startle testing and 24 new images. They pressed either the “new” or “old” button to indicate whether they had seen the image during startle testing. Recognition levels were 94.75% correct for Study 1.

##### Dependent measures

Raw EMG data sampled at 1000 Hz were smoothed by a software program (SR-HLAB Review Data, San Diego Instruments, San Diego, CA). Smoothing was accomplished by computing 3 ms rolling averages over the rectified and band-passed data (10–1000 Hz). Peak magnitude was defined as the point of maximal responding, provided the peak was a minimum of 2 standard deviations above baseline. Trials that failed to meet this threshold were retained and scored as having a zero-response magnitude. Trials were rejected if excessive activity was observed during the pre-startle baseline, which was defined as the 20 ms interval in advance of the presentation of the startle stimulus. Discarded trials comprised 8.2% of all trials in Study 1 and 12.6% in Study 2. Startle magnitudes were log (ln) transformed^80^ to meet the assumptions of parametric tests. This transformation minimized the effects of especially large startle responses. Non-responders were retained because of the nature of the hypothesis.

#### Data analysis

All statistical tests used a two-tailed rejection criterion of 0.05. The primary results were correlations between startle and the questionnaire measures. These correlational analyses were supplemented with multiple linear regression analyses to control for gender and identify any unique contributions of the trait predictors.

### Results and discussion

Negative images (*M* = 4.08) produced similar levels of startle responses as positive (*M* = 4.06) and neutral images (*M* = 4.09). Given the absence of a valence effect, *F*(2, 318) < 1.0, we did not examine ASP in Study 1. We did find somewhat lower startle magnitudes in the absence of a photo (*M* = 3.98) than when a photo was present, *F*(3, 477) = 7.82, *p* < 0.001, η_p_^2^ = 0.05. Bonferroni tests confirmed there was more startle when any image was present (positive, negative, or neutral) relative to when an image was absent. Figure S1 displays the mean levels of startle reactivity to the tactile and auditory stimuli as a function of trial block and gender.

#### General startle (GS)

Table 1 displays the bivariate correlations between the personality traits and general startle. The inter-correlations among the trait predictors themselves are found in Table S2. As predicted, sadism and GS were negatively correlated. Unexpectedly, none of the other Dark Tetrad traits predicted blunted GS. Regression analyses confirmed SD4 sadism had a unique association with blunted startle controlling for the other three members of the Dark Tetrad: Sadism predicted startle reactivity, β =  − 0.27, *p* = 0.003; whereas narcissism, β =  − 0.07, *p* = 0.43, Machiavellianism, β = 0.05, *p* = 0.57, and psychopathy, β = 0.00, *p* = 0.97, did not. When SD4 sadism scores were replaced by SSIS scores in the regression, a similar result emerged (β =  − 0.19, *p* = 0.04, for sadism; β =  − 0.07, *p* = 0.44, for narcissism, β = 0.02, *p* = 0.79, for Machiavellianism, and β =  − 0.03, *p* = 0.77, for psychopathy).

**Table 1:** Descriptive statistics (means, standard deviations) for the personality variables and bivariate correlations with self-reported startle and general startle in study 1*.*

Measure	*M*	*SD*	SRS	GS
1. SD4				
Machiavellianism	3.33	0.64	0.01	−0.06
Narcissism	2.98	0.66	−0.10	−0.11
Psychopathy	2.13	0.71	−0.18*	−0.13
Sadism	2.39	1.04	−0.29**	−0.27**
2. SSIS sadism	1.60	0.69	−0.14	−0.21*
3. Intolerance of uncertainty	2.81	0.80	0.34**	0.16*
4. Anxiety sensitivity	1.51	0.74	0.31**	0.14
Physical	1.30	0.89	0.25**	0.15
Cognitive	1.15	0.93	0.20**	0.06
Social	2.09	0.87	0.31**	0.13
5. BIS/BAS				
BIS	3.21	0.52	0.45**	0.20*
BAS drive	2.76	0.59	0.00	−0.01
BAS fun	3.06	0.56	−0.19	−0.13
BAS reward	3.61	0.32	0.11	0.10
6. Gender (M = 1, W = 2)			0.36**	0.28**

*N* = 160.

*SRS* self-reported startle, *GS* general startle, *SD4* Short Dark Tetrad, *SSIS* Short Sadistic Impulse Scale, *intolerance of uncertainty* Intolerance of Uncertainty Scale (IUS-12), *anxiety sensitivity* Anxiety Sensitivity Scale (ASI-3), *BIS/BAS* Behavioral Inhibition System/Behavioral Activation System, *M* men, *W* women.

*p < 0.05, **p < 0.01.

GS was greater for women (*M* = 4.12, *SD* = 0.78) than men (*M* = 3.64, *SD* = 0.63), *t*(91.39) =  − 4.01, *p* < 0.001, *d* = 0.68, as reported previously^81–83^. There was also a strong gender difference for SD4 sadism: men (*M* = 3.42, *SD* = 0.80) scored higher than women (*M* = 2.02, *SD* = 0.85), *t*(158) = 9.39, *p* < 0.001, *d* = 1.67 (also see Table 1). Multiple regression analysis found that SD4 sadism’s association with blunted startle did not survive a control for gender, β =  − 0.16, *p* = 0.10.

Finally, as in previous work^68, 69^, we found positive correlations between startle reactivity and anxiety/worry, as indexed by the BIS and IUS-12 (see Table 1). The ASI-3 correlation did not reach significance*, p* = 0.09.

#### Self-reported startle

Participants’ self-reports of their ease of being startled were positively correlated with GS, *r*(158) = 0.32, *p* < 0.001. As shown in Table 1, there was a remarkable degree of agreement between self-reported and lab-tested startle as a function of personality. For example, respondents with higher scores on the sadism subscale of the SD4 said they were not easily startled. By contrast, self-reported startle and GS levels were elevated when respondents reported higher levels of worry/anxiety on the IUS-12.

## Study 2

In Study 2, we aimed to replicate the negative association between sadism and general startle reactivity found in Study 1. This study examined personality predictors of three indexes of startle reactivity: GS, SRS, and ASP (i.e., a startle response potentiated by negative images). To induce intense affect at the arrival of the startle stimulus, we used normative data to arrange the images into movie-like sequences with increasing intensity, culminating in the last image paired with the startle stimulus (always a noise burst in Study 2). Successively displayed images were arranged in a series from near-neutral valence to intensely negative/positive. Finally, we expanded our questionnaires to include multifaceted measures of sadism^66^, psychopathy^64, 67^, and maladaptive traits associated with antagonistic behavior^73, 74^ to isolate the specific aspects of sadism and psychopathy that are most relevant to the observed effects. The primary aims of Study 2 were to (a) produce ASP with emotional images and (b) detect whether psychopathy and sadism were preferentially associated with blunted ASP.

### Method

#### Participants

In Study 2 (*N* = 244), Cohort 1 included 152 participants (112 women, 40 men) drawn without preselection from the introductory psychology subject pool. Cohort 2 included 92 participants (70 women, 22 men) preselected from the lowest or highest sadism quintile of the SD4 (included in a separate survey administered in September 2019). As in Study 1, data from the two cohorts were combined. Startle testing occurred from December 2019 to March 2020. Informed consent was obtained from all participants.

#### Measures

##### Personality Inventory for Diagnostic and Statistical Manual of Mental Disorders (PID-5)^73, 74^

The PID-5 assesses maladaptive traits involved in personality disorders. Facets measured were callousness, hostility, risk-taking, restricted affectivity, anxiousness, and emotional lability. The first four facets were selected for their relevance to sadism or psychopathy, while anxiousness and emotional lability were selected as indicators of startle-relevant negative affectivity. Participants rated the statements on a 4-point Likert scale ranging from 0 (*very false or often false*) to 3 (*very true or often true*). Cronbach’s alphas ranged from 0.82 to 0.90.

##### Self-Report Psychopathy Scale Short Form (SRP-SF)^67, 84^

The SRP-SF is a 29-item measure of adult psychopathic features with four subscales: interpersonal exploitation, callousness, lifestyle, and antisocial behavior. The SRP-SF offers a multifaceted view of psychopathic tendencies, complementing the SD4 psychopathy scale, which focuses on overall psychopathic tendencies. Items are rated on a 5-point Likert scale (1 = *disagree strongly* to 5 = *agree strongly*). The antisocial behavior subscale had a poor alpha in this sample (α = 0.39) and was not considered further. Cronbach’s alphas for the remaining subscales and total score were acceptable to good, 0.80 (SRP total), 0.84 (interpersonal), 0.67 (callousness), and 0.80 (lifestyle).

##### Self-Reported Startle (SRS)

Participants estimated their startle reactivity with a single item, “*Are you easily startled?*”, rated on a 5-point Likert scale with response options, 1 (*Not at all*), 2 (*A little bit*), 3 (*Somewhat*), 4 (*Very easily*), and 5 (*Extremely easily*).

##### Short Dark Tetrad (SD4)^5^

The SD4 is a 28-item measure of Machiavellianism, narcissism, psychopathy, and sadism. As in Study 1, we administered this measure twice: the first administration was done online and used only to identify the top and bottom quintiles; the second was conducted in person, alongside the other questionnaire measures, and was used to predict behavioral startle. Items were rated on a 5-point Likert scale from 1 (*strongly disagree*) to 5 (*strongly agree*). Cronbach’s alphas ranged from 0.78 to 0.87.

##### Short Sadistic Impulse Scale (SSIS)^65^

As in Study 1, we included the 10-item SSIS as an additional measure of overall sadism. Items were rated on a dichotomous scale, 0 (*unlike me*) to 1 (*like me*). Cronbach’s alpha was 0.58.

##### Triarchic Psychopathy Measure (TriPM)^64, 85^

The TriPM is a 58-item measure of psychopathy with three subscales: disinhibition (i.e., impulsive and antisocial tendencies), boldness (i.e., social dominance and stress immunity), and meanness (i.e., callousness and cold-hearted aggression)^64^. The TriPM measure of psychopathy, like the SRP-SF, partitions psychopathy into an array of component tendencies, providing complementary information to that obtained from the SD4 psychopathy scale. Items were rated on a 4-point Likert scale (response options: *true*, *somewhat true*, *somewhat false*, and *false*). Cronbach’s alphas ranged from 0.82 to 0.87.

##### Varieties of Sadistic Tendencies (VAST)^66^

The VAST is a 16-item measure of everyday sadism with subscales to assess direct sadism (enjoying inflicting suffering) and vicarious sadism (enjoying watching suffering). While these facets are highly related, they represent distinct aspects of sadistic tendencies that may exhibit divergent relationships with behavioral criteria. Items were rated on a 5-point Likert scale from 1 (*disagree strongly*) to 5 (*agree strongly*). Cronbach’s alphas were 0.80 (vicarious sadism), 0.64 (direct sadism), and 0.81 (VAST total).

#### Procedure

##### Startle testing

We used the same software and equipment as Study 1. The IAPS images used in Study 2 (see Table S3 of supplemental materials) were arranged into trials of eight successive images ordered by valence (ascending positive starting at *M* = 4.70 and ending at *M* = 8.20, descending negative starting at *M* = 4.02, and ending at *M* = 1.76, and unchanging neutral starting at *M* = 4.99 and ending at *M* = 4.94). Trials were separated by an average intertrial interval of 40 s. Each image was presented for 4 s, and the 20-ms, 105-dB startle stimulus began 2.0 s following the onset of the eighth image in the series. Trials were organized into three blocks of six trials each, positive (P), neutral (C = control), or negative (N). The order of the blocks was varied (PCN, PNC, NCP, NPC, CNP, and CPN) across participants to control for order effects. In total, participants received 16 startle probes (all auditory stimuli). There was no recognition test in this study.

#### Data analysis

All statistical tests used a two-tailed rejection criterion of 0.05. As in Study 1, the primary results were correlations between startle indexes and the questionnaire measures. The associations were further analyzed with multiple linear regression to control for gender and examine the unique contributions of the trait predictors.

### Results and discussion

Our attempt to produce ASP in Study 2 was successful. There was a main effect of valence, *F*(2, 486) = 14.63, *p* < 0.001, η_p_^2^ = 0.11. Bonferroni adjusted comparisons revealed higher log startle magnitudes during negative images (*M* = 6.30) than positive (*M* = 6.06) and neutral images (*M* = 6.11), which did not differ from each other. A difference score was computed to index ASP ([average amplitude for negative images] – [average amplitude of the positive and neutral images]). Figure S2 displays the mean levels of GS startle reactivity as a function of trial block and gender. Table S4 presents the intercorrelations among the trait predictors.

#### General startle (GS)

As shown in Table 2, all four SD4 subscales were negatively associated with GS, as were scores on two other psychopathy measures (TriPM and SRP) and VAST sadism. At the facet level, GS demonstrated negative correlations with vicarious sadism, as well as with two psychopathy subscales: SRP-SF interpersonal and TriPM boldness. In a multiple regression analysis, none of the SD4 measures emerged as unique predictors of GS (*p*s > 0.15), which suggests the empirical overlap in the Dark Tetrad traits (sometimes described as callous exploitation) accounts for their individual bivariate associations with blunted GS. Again, women were more easily startled (*M* = 6.43, *SD* = 0.86) than men (*M* = 5.73, *SD* = 0.98), *t*(242) =  − 5.30, *p* < 0.001, *d* = 0.78. All SD4 associations with GS dropped to non-significance when controlling for gender (*p*s > 0.18).

**Table 2:** Descriptive statistics (means, standard deviations) and bivariate correlations with self-reported startle, general startle, and aversive startle potentiation in study 2.

Measure	*M*	*SD*	SRS	GS	ASP
1. SD4					
Machiavellianism	3.26	0.77	−0.22**	−0.17**	−0.09
Narcissism	2.90	0.79	−0.21**	−0.13*	−0.11
Psychopathy	1.60	0.63	−0.30**	−0.18**	−0.18**
Sadism	2.18	1.00	−0.27**	−0.15*	−0.06
2. SSIS sadism	0.86	1.24	−0.18**	−0.03	−0.17**
3. VAST sadism	1.87	0.52	−0.39**	−0.15*	−0.21**
Vicarious	2.09	0.77	−0.35**	−0.15*	−0.16*
Direct	1.70	0.46	−0.34**	−0.11	−0.21**
4. SRP-SF psychopathy^a^	1.78	0.48	−0.26**	−0.15*	−0.20**
Interpersonal	1.86	0.75	−0.23**	−0.17**	−0.20**
Callousness	1.89	0.61	−0.26**	−0.11	−0.16*
Lifestyle	2.09	0.63	−0.21**	−0.11	−0.16*
5. TriPM psychopathy	0.94	0.30	−0.37**	−0.15*	−0.20**
Boldness	1.46	0.44	−0.34**	−0.17**	−0.12
Meanness	0.57	0.41	−0.39**	−0.10	−0.16*
Disinhibition	0.80	0.42	−0.07	−0.05	−0.13*
6. PID-5 select facets					
Anxiousness	1.86	0.78	0.19**	0.12	0.08
Callousness	0.35	0.37	−0.33**	−0.12	−0.20**
Emotional lability^b^	1.48	0.76	0.25**	0.06	0.01
Hostility	0.90	0.61	−0.05	0.01	−0.08
Restricted affect	0.81	0.74	−0.24**	−0.15*	−0.18**
Risk-taking	1.20	0.56	−0.26**	−0.13	−0.14*
7. Gender (M = 1, W = 2)			0.26**	0.32**	0.15*

*N* = 244.

*SRS* self-reported startle, *GS* general startle, *ASP* aversive startle potentiation, *SD4* Short Dark Tetrad, *SSIS* Short Sadistic Impulse Scale, *VAST* Varieties of Sadistic Tendencies, *SRP-SF* Self-Report Psychopathy Scale Short Form, *TriPM* Triarchic Psychopathy Measure, *PID-5* The Personality Inventory for Diagnostic and Statistical Manual of Mental Disorders Fifth Edition, *M* men, *W* women.

*p < 0.05, **p < 0.01.

^a^The antisocial behavior subscale was omitted due to poor alpha reliability (α = .39).

^b^*N* = 240 with four missing values.

#### Aversive startle potentiation (ASP)

Table 2 also shows an overall pattern of negative associations between ASP and measures of psychopathy and sadism, many of their facets, and related personality tendencies (callousness, risk-taking, and restricted affect). Sadistic tendencies assessed by the SSIS and VAST were significantly associated with blunted ASP, but unexpectedly, SD4 sadism was unrelated to ASP. By contrast, total scores on all psychopathy measures were associated with blunted ASP, as were all psychopathy facets except for TriPM boldness. Psychopathy was the only SD4 variable negatively associated with ASP, and the sole unique predictor of ASP (β =  − 0.21, *p* = 0.01) when all four SD4 subscales were entered in a multiple regression analysis.

As women had higher ASP indexes (*M* = 0.27, *SD* = 0.60) than men (*M* = 0.06, *SD* = 0.59), *t*(242) =  − 2.38, *p* = 0.02, *d* = 0.35, we also examined associations controlling for gender. Results of multiple regression analyses indicated that the significant sadism associations with blunted ASP survived a control for gender, SSIS β =  − 0.14, *p* = 0.03; and VAST β =  − 0.18, *p* = 0.02. The same was true of psychopathy scores, SD4 β =  − 0.15, *p* = 0.03; SRP-SF β =  − 0.17, *p* = 0.01; and TriPM β =  − 0.16, *p* = 0.02. Thus, the observed associations cannot be explained by mean gender differences.

Additional analyses were performed to tease apart the effect of direct and vicarious sadism. When vicarious sadism, direct sadism, and gender were entered into a regression, only direct sadism emerged as a significant unique predictor of ASP, β =  − 0.17, *p* = 0.02. Direct sadism measures behavioral sadistic tendencies (e.g., “I enjoy mocking losers to their face”) as opposed to vicarious sadism (e.g., “In video games, I like the realistic blood spurts”). Direct sadism is an escalatory measure in the sense it involves both the participation and enjoyment of hurtful activities.

The PID-5 facets of callousness, restricted affect, and risk-taking correlated negatively with ASP (see Table 2). By contrast, hostility, anxiousness, and emotional lability were uncorrelated with ASP. The failure of hostility in that analysis suggests not all antagonistic traits predict blunted startle^86^, particularly if they include a strong negative affectivity component that might offset or oppose the startle-dampening effects of its antagonistic component. In regression analyses with gender entered as a covariate, callousness, β =  − 0.17, *p* = 0.01, and restricted affect, β =  − 0.16, *p* = 0.02, remained associated with reduced ASP controlling for gender, but risk-taking did not, β =  − 0.11, *p* = 0.10.

#### Self-reported startle

Subjective estimates of startle reactivity correlated positively with both GS, *r*(242) = 0.17, *p* = 0.01, and ASP, *r*(242) = 0.16, *p* = 0.01, but GS and ASP were uncorrelated with each other, *r*(242) =  − 0.04, *p* = 0.56. Table 2 shows a good agreement between trait associations of self-reported startle and trait associations of lab-tested startle with a few minor exceptions.

## General discussion

We evaluated the relationship between the Dark Tetrad and startle reactivity in an undergraduate university student population. The novel finding was that ordinary people with sadistic tendencies display the same biomarker previously reported for psychopathic tendencies^35^. In both studies, people with sadistic traits showed blunted general startle, averaged across positive, neutral, and negative images. In Study 2, sadism was associated with blunted ASP, suggesting immunity to startle potentiation by negatively-valenced images. Relative to low scorers, participants with high scores on the VAST and SSIS sadism measures did not startle as strongly while viewing unpleasant images (e.g., depicting threats, suffering, accidents, and other distasteful scenes) than pleasant and neutral images. In line with past findings^36–38, 62, 87^, psychopathy correlated negatively with ASP, as did the related pathological tendencies of callousness, restricted affect, and risk-taking. By contrast, Machiavellianism and narcissism (the two remaining members of the Dark Tetrad) were not individually associated with ASP, despite their moderate correlations with sadism and psychopathy. Overall, the results of these studies suggest that startle reactivity (particularly ASP) varies as a function of subclinical sadism and psychopathy. The findings of these studies confirm the importance of sadism in predicting individual differences in startle responses. They also highlight the need for further research on the physiological underpinnings of sadistic tendencies.

Our results are consistent with the bifactor model proposed by Anderson and Marcus^88^, in which antagonism (defined as low agreeableness on the five-factor model) underlies both psychopathy and sadism, but sadism involves the additional factor of deriving pleasure from the suffering of others. We found similar effects of sadism and psychopathy on ASP. However, the underlying mechanisms may differ. Blunted ASP in everyday sadism may be due to pleasure-induced attenuation and/or antagonism, while blunted ASP in everyday psychopathy may be due to antagonism alone. If the sadism effect involves pleasure from aversive images, then sadistic tendencies may be particularly effective at disrupting startle responses, which tend to be attenuated by positive affect^33^. On the other hand, an antagonistic orientation may be sufficient on its own.

Beyond general antagonism, the specific traits of callous unemotionality^89–91^ and fearless dominance^92^ are the two personality factors/facets most consistently linked to diminished ASP in psychopathy^35, 62^. These traits are closely related to the meanness and boldness factors of the Triarchic Model of Psychopathy^64, 85, 93^, which are also negatively correlated with ASP^35^. In one study, meanness predicted blunted ASP in adulthood, controlling for adolescent-assessed narcissism and impulsivity^91^. Other research documents negative associations between boldness and ASP controlling for meanness^94^, suggesting that psychopathy facets may have complex relationships with startle reactivity. In our research, boldness was associated with blunted GS, whereas meanness was associated with blunted ASP. The findings suggest that psychopathic boldness and meanness may operate somewhat differently on startle reactivity; however, they both are thought to reflect genotypic under-reactive defensive systems.

These two psychopathy factors, fearless dominance/boldness and callousness/cold-heartedness, correspond to locations that are orthogonal in the Interpersonal Circumplex Model (IPC)^95^. This model holds that the fundamental social meaning of all important interpersonal behavior styles is well-described by a two-dimensional circular continuum^96^, organized around the orthogonal dimensions of assured/dominant behavior (dominance axis) and warm/agreeable behavior (warmth axis). The model could suggest two unrelated startle effects—one linked to social boldness (IPC 90 degrees) and one linked to callousness (IPC 180 degrees)—or a common startle effect associated with a personality vector midway between those factors^97^. On the IPC model, however, that midway location corresponds to “calculating” or manipulative traits, leading to the questionable prediction that Machiavellianism should be the strongest predictor of ASP. Thus, the results of past studies seem best explained by the independent effects of callousness (or cold-heartedness) and fearless dominance (or boldness)^35^.

Gender is another important predictor of startle reactivity. In both studies, we found significant gender differences in sadism and startle responses that required additional analyses to disentangle their effects. In Study 1, sadism failed to emerge as an incremental predictor of GS beyond overlap with gender differences. However, in Study 2, blunted ASP was associated with scores on most measures of psychopathy, sadism, and associated tendencies when controlling for gender. Although further research is merited, our data suggest that it is unlikely that gender differences in startle explain the link between dark traits and ASP. Indeed, much like men with psychopathic traits, women with psychopathic traits show diminished startle responses when viewing unpleasant pictures^41, 98^, which suggests gender on its own is probably not the sole explanation.

In both studies, self-reported startle estimates converged with the experimental startle magnitudes (GS and ASP). However, despite their similarities, GS and ASP were uncorrelated with each other. In other words, high levels of GS do not necessarily correspond to high levels of ASP, and vice versa. These two measures capture distinct aspects of the startle response^99^. The eyeblink reflex is a brain stem reaction to sensory input that activates spinal and motor neurons; however, it is modulated by higher brain states. The key interface identified in animal studies is the caudal pontine reticular nucleus^100, 101^, which receives a direct projection from the central nucleus of the amygdala, and indirect projections from the deep mesencephalic reticular formation, ventral periaqueductal gray, and the ventromedial hypothalamus. These direct and indirect projections may inhibit or potentiate the startle reflex, depending on experimental circumstances (e.g., viewing emotional stimuli). The affective context moderates the individual’s reflexive startle response, which itself could be weaker or stronger.

GS is a measure of overall startle reactivity that statistically controls for image valence, producing an index of startle reactivity that is independent of the affective context. ASP, on the other hand, focuses on startle reactivity heightened by negative affect, compared to positive/neutral affect. Because GS and ASP evaluate different aspects of startle reactivity, they tend to show different patterns of association with individual difference variables. For example, neuroticism and depression are often associated with increased GS, but unrelated to ASP^99^. In our research, gender was an important correlate of GS in both Study 1 and 2, whereas dark personality variables played a larger role in ASP, with independent contributions controlling for gender in Study 2. Presumably, individuals with high levels of sadism and psychopathy view negative images less negatively (or even positively, in the case of sadism) than individuals with lower levels of these traits^102^, irrespective of gender. Future research should continue to explore the trait correlates of both GS and ASP measures of startle reactivity. Researchers should also consider splitting the aversive stimuli into specific categories (e.g., fear, suffering, disgust, etc.) to generate high-fidelity reactivity profiles for each Dark Tetrad trait. This approach could provide valuable insights into the mechanisms underlying blunted ASP in sadism and psychopathy.

### Limitations

One surprising finding was that the SD4 sadism scale was uncorrelated with ASP in Study 2, despite its strong associations with other sadism measures (VAST and SSIS) that correlated negatively with ASP. These differences may be explained by differences in how these scales assess sadistic tendencies. The SD4 sadism scale is a brief screening measure with broader content and softer, more everyday indicators than the SSIS^5^. Thus ASP may relate most strongly to severe sadistic tendencies involving pleasure from hurting others or witnessing profound suffering. This relationship between ASP and sadistic tendencies may be more pronounced when using a comprehensive and multifaceted questionnaire to assess sadism, rather than a unidimensional screening measure like the SD4 sadism subscale.

Our reliance on the affective impact of single IAPS images may have limited findings in Study 1. Previous research has documented age-related differences in ratings of valence and arousal^103^: younger adults perceive negative images as less negative and less arousing, and positive images as less positive and more arousing, compared to older adults. Since most of our participants were young undergraduate students, the affective impact of the negative images may not have been strong enough to support ASP in Study 1. Although Studies 1 and 2 differed in a number of ways, Study 2 used an escalating series of IAPS images that culminated in the most positive or negative image. This procedure may have been more effective in eliciting ASP. Our results suggest that arranging normed images into more impactful, movie-like sequences can mitigate some of the age-related issues with emotional images, and allow for a more nuanced examination of ASP associations with personality variables and different image categories. Another positive development is the growing availability of emotional video clip databases^104^.

The significant associations between self-reported startle estimates and GS/ASP require some care in their interpretation. The most straightforward interpretation is that people have observed others being startled, and have experienced their own startle responses, permitting a comparison. Another possibility is that people have a stereotyped view of the kinds of individuals who startle easily (e.g., women or highly sensitive people) or those who do not (e.g., hypermasculine men). This could lead them to assume these stereotypes apply to themselves. Additional research is needed to determine the extent to which people have accurate self-knowledge about their startle tendencies and the best method to assess these tendencies with self-report measures.

Past research has often shown that implicit measures correlate better with other implicit measures, while self-report measures correlate better with other self-report measures^105^. Thus, future research could test startle associations with more observational or implicit measures of antisocial traits, such as the Interpersonal Measure of Psychopathy^106^, the Psychopathy Q-Set^107^, the Revised Animal Preference Test^108^, the voodoo doll aggression task^109^, or tasks to assess implicit associations with violence^110, 111^. Informant reports of sadistic tendencies may also help corroborate the self-reports and provide additional information from an observer’s perspective.

## Conclusion

While previous research demonstrates a relationship between psychopathy and blunted startle reactivity in forensic and community samples, the present studies are the first to focus on everyday sadism. In Study 1, sadism was an incremental predictor of general startle reactivity beyond the other traits of the Dark Tetrad. In Study 2, both psychopathy and sadism, and associated pathological traits, predicted blunted startle potentiation by aversive images, while the Dark Tetrad traits were associated with blunted startle in general. We conclude that individuals with high levels of sadism show a diminished startle reflex that is relatively immune to potentiation by negative environmental stimuli. These findings provide further insight into the biological markers of the Dark Tetrad traits and their unique facets. Our findings may also have implications for fields beyond psychology, like business and economics, where managerial effectiveness (e.g., navigating workplace crises) and financial decision-making (e.g., loss aversion and risk-taking) may depend on the personality of a single individual with socially aversive tendencies. Further investigation should contribute to a deeper understanding of the Dark Tetrad traits and inform strategies for mitigating their impact in everyday life.

### Supplementary Information

Supplementary Information.

## Data Availability

Data and supplemental materials are available for download on the Open Science Framework (https://osf.io/wh6zr).
