# Supplementary material for: Blunted startle reactivity in everyday sadism and psychopathy
Source: Sci Rep. 2023 Aug 30;13:14216. doi: 10.1038/s41598-023-41043-2 (PMC10469178; doi:10.1038/s41598-023-41043-2)
Supplement: Supplementary file 1 — Supplementary Information. [file 41598_2023_41043_MOESM1_ESM.docx]

Supplemental Materials

Buckels, E. E., Williams, D. A., Trapnell, P. D., Koosheh, S. K., Javra, O. M., & Svenne, S. C.
Blunted startle reactivity in everyday sadism and psychopathy.

**Table S1**

*Study 1 IAPS Images*

| Positive Images | IAPS Image | Valence | Neutral Images | IAPS Image | Valence | Negative Images | IAPS Image | Valence |
| --- | --- | --- | --- | --- | --- | --- | --- | --- |
| Athletes | 8540 | 7.47 | Abstract Art | 7237 | 5.43 | Accident | 3015 | 1.59 |
| Baby | 2050 | 8.21 | Bakers | 2579 | 5.55 | Accident | 9435 | 2.30 |
| Bunnies | 1740 | 6.91 | Bird | 1616 | 5.24 | Attack Dog | 1525 | 3.11 |
| Ice Cream | 7330 | 7.63 | Boat | 5395 | 5.34 | Bloody Kiss | 2352.2 | 2.14 |
| Kitten | 1460 | 8.19 | Book | 7090 | 5.20 | Cigarettes | 9830 | 2.54 |
| Money | 8502 | 7.49 | Fire Hydrant | 7100 | 5.25 | Duck in Oil | 9560 | 2.13 |
| Money | 8501 | 7.91 | Flowers | 5731 | 5.39 | Hospital | 2205 | 1.95 |
| Mother | 2311 | 7.53 | Fork | 7080 | 5.27 | Hospital | 3220 | 2.50 |
| Puppies | 1710 | 8.31 | Fruit | 7283 | 5.50 | KKK Rally | 9810 | 2.10 |
| Sailing | 8080 | 7.73 | Hair Dryer | 7050 | 4.93 | Man In Pool | 2055.1 | 3.18 |
| Seagulls | 5831 | 7.56 | Mushroom | 5500 | 5.42 | Roaches | 1274 | 3.18 |
| Seal | 1440 | 8.20 | Musician | 2487 | 5.19 | Skinhead | 9800 | 2.06 |
| Skier | 8190 | 8.11 | Plant | 5740 | 5.20 | Soldier | 9421 | 2.22 |
| Sky | 5982 | 7.62 | Ramen | 7490 | 5.49 | Toddler | 2095 | 1.82 |
| Sunset | 5830 | 7.96 | Shopping | 2745.1 | 5.30 | Toilet | 9301 | 2.30 |
| Water Slide | 8496 | 7.52 | Tourist | 2850 | 5.19 | War | 2683 | 2.65 |
|  |  |  |  |  |  |  |  |  |

*Note.* IAPS = International Affective Picture System. Valence ratings from Lang et al. (2008).

| **Table S2**  *Inter-correlations Between Predictor Variables in Study 1* | | | | | | | | | | | | | | |
| --- | --- | --- | --- | --- | --- | --- | --- | --- | --- | --- | --- | --- | --- | --- |
| Variable | 1 | 2 | 3 | 4 | 5 | 6 | 7 | 8 | 9 | 10 | 11 | 12 | 13 | 14 |
| SD4 |  |  |  |  |  |  |  |  |  |  |  |  |  |  |
| 1. Machiavellianism | (.72) |  |  |  |  |  |  |  |  |  |  |  |  |  |
| 2. Narcissism | .27^**^ | (.74) |  |  |  |  |  |  |  |  |  |  |  |  |
| 3. Psychopathy | .32^**^ | .30^**^ | (.77) |  |  |  |  |  |  |  |  |  |  |  |
| 4. Sadism | .33^**^ | .23^**^ | .48^**^ | (.87) |  |  |  |  |  |  |  |  |  |  |
| 5. SSIS | .29^**^ | .25^**^ | .48^**^ | .58^**^ | (.88) |  |  |  |  |  |  |  |  |  |
| 6. IUS-12 | .16^*^ | .00 | -.09 | -.20^*^ | -.01 | (.89) |  |  |  |  |  |  |  |  |
| 7. ASI-3 | .07 | -.01 | -.01 | -.22^**^ | -.09 | .59^**^ | (.90) |  |  |  |  |  |  |  |
| 8. Physical | .06 | -.03 | .03 | -.17^*^ | -.02 | .41^**^ | .83^**^ | (.82) |  |  |  |  |  |  |
| 9. Cognitive | .18^*^ | .14 | .06 | -.16^*^ | .00 | .47^**^ | .89^**^ | .66^**^ | (.87) |  |  |  |  |  |
| 10. Social | -.06 | -.13 | -.10 | -.22^**^ | -.22^**^ | .59^**^ | .77^**^ | .41^**^ | .54^**^ | (.75) |  |  |  |  |
| BIS/BAS |  |  |  |  |  |  |  |  |  |  |  |  |  |  |
| 11. BIS | -.05 | -.13 | -.22^**^ | -.23^**^ | -.21^**^ | .53^**^ | .36^**^ | .25^**^ | .22^**^ | .43^**^ | (.80) |  |  |  |
| 12. Drive | .22^**^ | .40^**^ | .16^*^ | .03 | .12 | .08 | -.11 | -.12 | -.03 | -.13 | -.05 | (.67) |  |  |
| 13. Fun | .16^*^ | .28^**^ | .26^**^ | 22^**^ | .12 | -.17^*^ | -.10 | -.10 | .00 | -.15 | -.13 | .38^**^ | (.70) |  |
| 14. Reward | .11 | .26^**^ | -.05 | -.08 | -.15 | .27^**^ | .06 | .05 | .02 | .08 | .30^**^ | .25^**^ | .17^*^ | (.73) |
| Gender (M=1, W=2) | -.17^*^ | -.12 | -.17^*^ | -.60^**^ | -.43^**^ | .20^*^ | .24^**^ | .22^**^ | .18^*^ | .21^**^ | .33^**^ | .05 | -.06 | .22^**^ |

*Note. N =* 160. * *p* < .05, ** *p* < .01. () = Cronbach’s Alpha, SD4 = Short Dark Tetrad, SSIS = Short Sadistic Impulse Scale, ASI-3 = Anxiety Sensitivity Scale, IUS-12 = Intolerance of Uncertainty Scale, BIS/BAS = Behavioral Inhibition System/Behavioral Activation System, M=men, W=women.

**Table S3**

*Study 2 IAPS Images*

| IAPS Image | | | | | | | | IAPS Valence | | | | | | | |
| --- | --- | --- | --- | --- | --- | --- | --- | --- | --- | --- | --- | --- | --- | --- | --- |
| 1 | 2 | 3 | 4 | 5 | 6 | 7 | 8 | 1 | 2 | 3 | 4 | 5 | 6 | 7 | 8 |
| Positive |  |  |  |  |  |  |  |  |  |  |  |  |  |  |  |
| 1350 | 1313 | 1410 | 1500 | 1463 | 5910 | 1441 | 1440 | 5.17 | 5.61 | 6.98 | 7.25 | 7.46 | 7.79 | 7.93 | 8.20 |
| 2309 | 1942 | 2250 | 1540 | 1999 | 5982 | 2080 | 1460 | 4.94 | 6.25 | 6.67 | 7.16 | 7.43 | 7.62 | 8.01 | 8.19 |
| 2411 | 2032 | 5870 | 1630 | 2209 | 7200 | 2340 | 1710 | 5.08 | 5.48 | 6.76 | 7.07 | 7.54 | 7.64 | 8.00 | 8.31 |
| 2520 | 2373 | 7325 | 4610 | 2341 | 7330 | 5760 | 2050 | 4.13 | 6.95 | 6.98 | 7.25 | 7.30 | 7.63 | 8.05 | 8.21 |
| 2795 | 2521 | 7340 | 7430 | 5600 | 7502 | 5825 | 5833 | 3.93 | 5.74 | 6.64 | 7.12 | 7.55 | 7.73 | 8.04 | 8.21 |
| 2890 | 7506 | 8503 | 8350 | 8502 | 8420 | 5830 | 8190 | 4.95 | 5.40 | 7.02 | 7.16 | 7.49 | 7.76 | 7.96 | 8.11 |
| Neutral |  |  |  |  |  |  |  |  |  |  |  |  |  |  |  |
| 6150 | 7004 | 5533 | 5130 | 7019 | 5395 | 7000 | 7014 | 5.09 | 5.02 | 5.31 | 4.45 | 5.23 | 5.34 | 5.00 | 5.14 |
| 7003 | 7006 | 7058 | 5471 | 9031 | 7011 | 7002 | 7059 | 5.00 | 4.87 | 5.38 | 5.23 | 2.98 | 4.55 | 4.97 | 4.94 |
| 7012 | 7030 | 7060 | 7046 | 7061 | 7018 | 7050 | 7179 | 4.99 | 4.70 | 4.44 | 4.19 | 5.41 | 4.85 | 4.93 | 5.06 |
| 7025 | 7077 | 7150 | 7062 | 7211 | 7052 | 7057 | 7235 | 4.63 | 5.19 | 4.73 | 5.21 | 4.84 | 5.35 | 5.35 | 4.96 |
| 7056 | 7500 | 7512 | 7100 | 7224 | 7165 | 7175 | 7491 | 5.05 | 5.34 | 5.42 | 5.25 | 4.45 | 6.04 | 4.87 | 4.83 |
| 7090 | 7632 | 7547 | 7217 | 7710 | 7705 | 7255 | 9468 | 5.20 | 5.19 | 5.21 | 4.82 | 5.41 | 4.77 | 5.06 | 4.69 |
| Negative |  |  |  |  |  |  |  |  |  |  |  |  |  |  |  |
| 2695 | 2751 | 6312 | 3181 | 2352.2 | 2205 | 3180 | 3005.1 | 4.02 | 2.78 | 2.48 | 2.40 | 2.14 | 1.95 | 1.97 | 1.66 |
| 6840 | 3185 | 6570 | 6021 | 2811 | 6313 | 3301 | 6563 | 3.75 | 2.91 | 2.20 | 2.32 | 2.29 | 2.02 | 1.91 | 1.87 |
| 9150 | 6831 | 7380 | 6230 | 3195 | 6520 | 3530 | 9040 | 4.58 | 2.60 | 2.46 | 2.40 | 2.18 | 2.02 | 1.81 | 1.69 |
| 9402 | 8230 | 9050 | 6821 | 3500 | 9254 | 9187 | 9075 | 4.48 | 3.14 | 2.48 | 2.41 | 2.22 | 2.08 | 2.00 | 1.79 |
| 9445 | 9265 | 9250 | 9300 | 6212 | 9414 | 9325 | 9183 | 4.04 | 2.64 | 2.60 | 2.37 | 2.20 | 2.12 | 1.96 | 1.74 |

*Note.* IAPS = International Affective Picture System. Column numbers indicate the ordinal position of the IAPS image (left-hand side) or its associated valence (right-hand side) on positive, neutral, and negative trials. On each trial, one of the six available slides for each ordinal position was presented randomly without replacement. Valence ratings from Lang et al. (2008).

**Table S4**

*Inter-correlations Between Predictor Variables in Study 2*

| Variable | 1 | 2 | 3 | | 4 | | 5 | 6 | 7 | 8 | | 9 | | 10 | | 11 | | 12 | | 13 | | 14 | | 15 | | 16 | | 17 | | 18 | | 19 | | 20 | | 21 | | 22 | |  |
| --- | --- | --- | --- | --- | --- | --- | --- | --- | --- | --- | --- | --- | --- | --- | --- | --- | --- | --- | --- | --- | --- | --- | --- | --- | --- | --- | --- | --- | --- | --- | --- | --- | --- | --- | --- | --- | --- | --- | --- | --- |
| SD4 |  |  |  | |  | |  |  |  |  | |  | |  | |  | |  | |  | |  | |  | |  | |  | |  | |  | |  | |  | |  | |  |
| 1. Machiavellianism | (.79) |  |  |  | |  | |  |  | |  | |  | |  | |  | |  | |  | |  | |  | |  | |  | |  | |  | |  | |  | |  | |
| 2. Narcissism | .38** | (.78) |  |  | |  | |  |  | |  | |  | |  | |  | |  | |  | |  | |  | |  | |  | |  | |  | |  | |  | |  | |
| 3. Psychopathy | .37** | .35** | (.79) |  | |  | |  |  | |  | |  | |  | |  | |  | |  | |  | |  | |  | |  | |  | |  | |  | |  | |  | |
| 4. Sadism | .48** | .28** | .60** | (.87) | |  | |  |  | |  | |  | |  | |  | |  | |  | |  | |  | |  | |  | |  | |  | |  | |  | |  | |
| 5. SSIS | .29** | .24** | .48** | .50** | | (.58) | |  |  | |  | |  | |  | |  | |  | |  | |  | |  | |  | |  | |  | |  | |  | |  | |  | |
| 6. VAST | .48** | .33** | .64** | .81** | | .51** | | (.81) |  | |  | |  | |  | |  | |  | |  | |  | |  | |  | |  | |  | |  | |  | |  | |  | |
| 7. Vicarious sadism | .37** | .24** | .52** | .80** | | .39** | | .91** | (.80) | |  | |  | |  | |  | |  | |  | |  | |  | |  | |  | |  | |  | |  | |  | |  | |
| 8. Direct sadism | .48** | .37** | .61** | .60** | | .53** | | .84** | .52** | | (.64) | |  | |  | |  | |  | |  | |  | |  | |  | |  | |  | |  | |  | |  | |  | |
| 9. SRP-SF | .59** | .37** | .69** | .74** | | .54** | | .77** | .62** | | .75** | | (.80) | |  | |  | |  | |  | |  | |  | |  | |  | |  | |  | |  | |  | |  | |
| 10. Interpersonal | .62** | .35** | .57** | .60** | | .50** | | .65** | .48** | | .69** | | .88** | | (.84) | |  | |  | |  | |  | |  | |  | |  | |  | |  | |  | |  | |  | |
| 11. Callousness | .50** | .31** | .59** | .77** | | .48** | | .74** | .66** | | .63** | | .86** | | .69** | | (.67) | |  | |  | |  | |  | |  | |  | |  | |  | |  | |  | |  | |
| 12. Lifestyle | .44** | .31** | .65** | .59** | | .39** | | .61** | .49** | | .59** | | .84** | | .61** | | .65^**^ | | (.80) | |  | |  | |  | |  | |  | |  | |  | |  | |  | |  | |
| 13. TriPM | .54** | .54** | .69** | .64** | | .55** | | .70** | .57** | | .67** | | .77** | | .63** | | .68^**^ | | .71** | | (.87) | |  | |  | |  | |  | |  | |  | |  | |  | |  | |
| 14. Boldness | .26** | .68** | .32** | .24** | | .22** | | .28** | .24** | | .26** | | .30** | | .23** | | .24^**^ | | .28** | | .59** | | (.82) | |  | |  | |  | |  | |  | |  | |  | |  | |
| 15. Meanness | .55** | .31** | .59** | .73** | | .54** | | .77** | .65** | | .70** | | .75** | | .66** | | .73^**^ | | .62** | | .82** | | .26** | | (.83) | |  | |  | |  | |  | |  | |  | |  | |
| 16. Disinhibition | .35** | .14* | .56** | .40** | | .41** | | .44** | .32** | | .46** | | .59** | | .45** | | .48^**^ | | .59** | | .71** | | -.01 | | .50** | | (.87) | |  | |  | |  | |  | |  | |  | |
| PID-5 Selected Facets |  |  |  |  | |  | |  |  | |  | |  | |  | |  | |  | |  | |  | |  | |  | |  | |  | |  | |  | |  | |  | |
| 17. Anxiousness | .06 | -.17** | .02 | .04 | | .01 | | . 00 | -.05 | | .07 | | .10 | | .07 | | .07 | | .11 | | -.03 | | -.40** | | .00 | | .34** | | (.90) | |  | |  | |  | |  | |  | |
| 18. Callousness | .51** | .32** | .57** | .55** | | .50** | | .69** | .52** | | .70** | | .69** | | .65** | | .64** | | .52** | | .71** | | .24** | | .79** | | .48** | | . 07 | | (.82) | |  | |  | |  | |  | |
| 19. Emotional Lability | .06 | -.05 | .13* | -.02 | | .10 | | -.06 | -.10 | | .01 | | .08 | | .03 | | .01 | | .15* | | .05 | | -.26** | | -.04 | | .38** | | .54** | | .07 | | (.83) | |  | |  | |  | |
| 20. Hostility | .40** | .23** | .52** | .49** | | .45** | | .47** | .33** | | .52** | | .57** | | .51** | | .51** | | .48** | | .59** | | .07 | | .58** | | .61** | | .32** | | .60** | | .35** | | (.85) | |  | |  | |
| 21. Restricted Affect | .39** | .25** | .41** | .36** | | .20** | | .42** | .34** | | .39** | | .47** | | .45** | | .52** | | .32** | | .50** | | .21** | | .50** | | .35** | | -.02 | | .50** | | -.15* | | .40** | | (.89) | |  | |
| 22. Risk-taking | .29** | .27** | .61** | .50** | | .36** | | .52** | .46** | | .44** | | .53** | | .34** | | .44** | | .66** | | .63** | | .41** | | .53** | | .39** | | -.08 | | .39** | | .06 | | .31** | | .30** | | (.89) | |
| Gender (M=1, W=2) | -.34** | -.18** | -.32** | -.51** | | -.21** | | -.54** | -.56** | | -.36** | | -.37** | | -.37** | | -.34** | | -.26** | | -.35** | | -.26** | | -.37** | | -.11 | | .09 | | -.29** | | .21** | | -.11 | | -.17** | | .27** | |

*Note. N =* 244, * *p* <.05, ** *p* < .01, () = Cronbach’s Alpha, SRS = self-reported startle, GS = general startle, ASP = aversive startle potentiation, SD4 = Short Dark Tetrad, SSIS = Short Sadistic Impulse Scale, VAST = Varieties of Sadistic Tendencies, SRP-SF = Self-Report Psychopathy Scale Short Format, TriPM = Triarchic Psychopathy Measure, PID-5 = The Personality Inventory for Diagnostic and Statistical Manual of Mental Disorders Fifth Edition, M=men, W=women.

**Figure S1**

*Mean Startle Reactivity to the Tactile and Auditory Stimuli as a Function of Trial Block and Gender in Study 1*


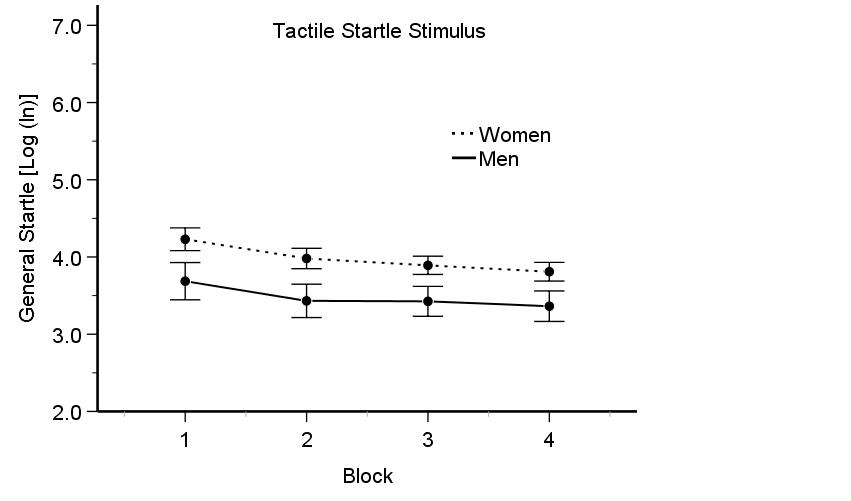

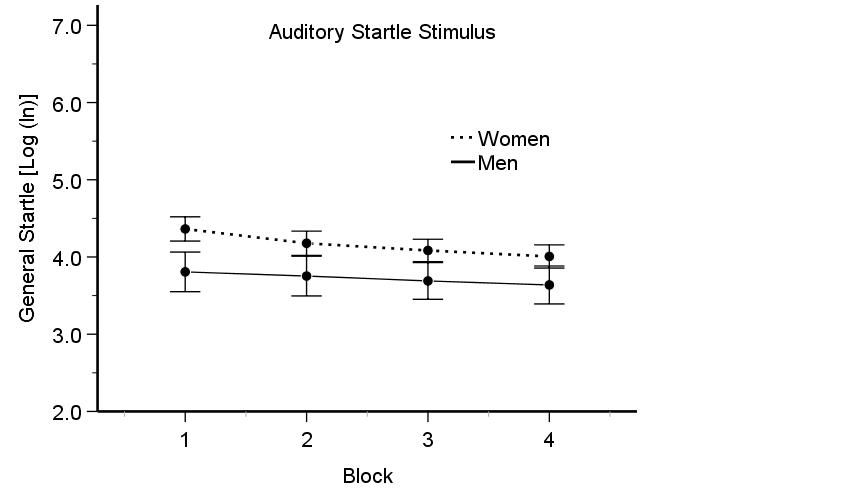


*Note.* Average startle levels per block to the tactile (air, left panel) and auditory (noise, right panel) startle stimuli in Study 1. Error bars are 95% confidence intervals. A 2 (startle stimulus) × 4 (block) × 2 (gender) ANOVA on the natural log transformed [Log (ln)] data revealed main effects of startle stimulus, *F*(1, 158) = 25.64, *p* < .001, block, *F*(3, 474) = 37.38, *p* < .001, and gender, *F*(1, 158) = 28.15, *p* < .001. No interactions were found. The block effect was due to habituation to both startle stimuli over repeated presentations within the startle testing session (startle stimuli balanced within blocks, 32 trials with 16 each of the air and noise). No ASP was found Study 1.

**Figure S2**

*Mean Startle Reactivity as a Function of Trial Block and Gender in Study 2*


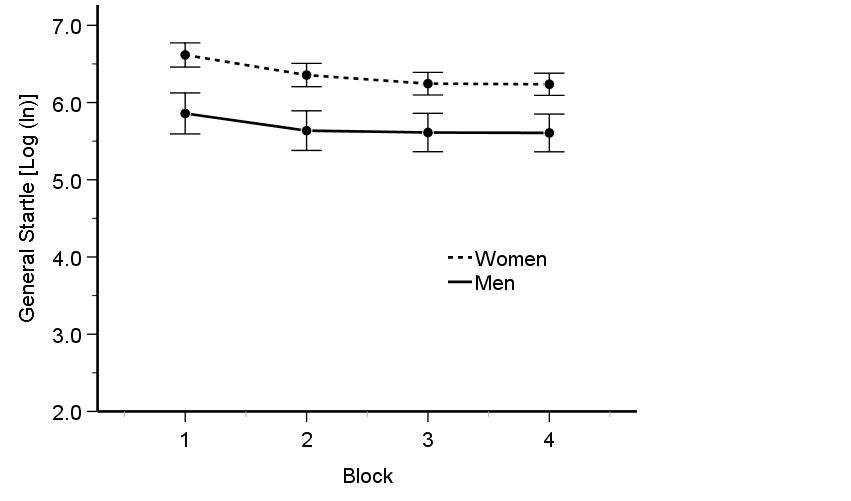


*Note.* Average startle levels to the noise startle stimulus in Study 2 (error bars are 95% confidence intervals). A 4 (block) × 2 (gender) ANOVA on the natural log transformed [Log (ln)] data revealed main effects of block, *F*(3, 726) = 15.29, *p* < .001, and gender, *F*(1, 242) = 25.98, *p* < .001. Block and gender did not interact. The block effect was due to habituation with repeated presentations of the noise startle stimulus over 16 trials. ASP was found Study 2.
